# Application of machine learning in predicting of MB2 canal in permanent maxillary first and second molars: A CBCT study

**DOI:** 10.1371/journal.pone.0357808

**Published:** 2026-09-09

**Authors:** Ranjdar Mahmood Talabani

**Affiliations:** Department of Operative Dentistry and Endodontics, College of Dentistry, University of Sulaimani, Sulaimani, Iraq; Universidade Federal Fluminense, BRAZIL

## Abstract

**Objectives:**

The aim of this study is to apply Machine learning (ML), a key subset of Artificial Intelligence (AI) in predicting second mesiobuccal (MB2) canals in both maxillary first molars (MFMs) and maxillary second molars (MSMs) using the cone-beam computed tomography (CBCT) imaging in an Iraqi sub-population.

**Methods:**

In this retrospective study, 144 CBCT scans from 63 female and 81 male patients were retrieved from the archives of a radiology department at B&R Dental Center. The presence and absence of MB2 canal in both MFMs and MSMs in relation to age, sex, and side, area of triangle (A) between MB, DB and P canals, semiperimeter (SP), and the distances between the orifices of mesio-buccal (MB), disto-buccal (DB), and palatal (P) canals in both MFMs and MSMs in the axial plane were measured on CBCT scans. Descriptive and inferential statistics were employed for data analysis. The receiver operating characteristic (ROC) curve analysis was employed to assess the diagnostic accuracy of all data in predicting the existence of an MB2 canal in both molars. The optimal cut-off point was established based on sensitivity and specificity. The models’ classification measures, including area under the curve (AUC), accuracy, F1-score, and precision, were evaluated.

**Results:**

Overall, the prevalence of the MB2 canal was 65.24% in maxillary first molars (MFMs) and 36.89% in maxillary second molars (MSMs). A significantly higher prevalence of the MB2 canal among females was observed only in MSMs (p < 0.001), whereas no significant sex difference was found in MFMs (p = 0.915). Logistic regression (LR) demonstrated the best performance among all machine learning (ML) models, achieving an AUC of 0.88 for MFMs and 0.90 for MSMs. Feature importance analysis using Random Forest (RF) and Decision Tree (DT) models identified the mesiobuccal-to-palatal (MB–P) distance as the most influential predictor of MB2 canal presence.

**Conclusions:**

The present study showed promising results in the ML based predicting of MB2 canal in both MFMs and MSMs using axial CBCT slices based on MB-P distance parameter as the most influential predictor for the presence of the MB2 canal in both MFMs and MSMs using all ML models. These findings might allow clinicians to identify MB2 canals in maxillary molars for effective endodontic treatment.

## 1. Introduction

The primary goal of endodontic treatment is thorough cleaning of the entire root canal system both mechanically and chemically, followed by obturation with an inert filling material [1]. One potential source of persistent endodontic infection, particularly in maxillary first and second molars, is the failure to locate and treat the entire root canal system during the initial root canal procedure [2]. Second mesiobuccal (MB2) canals can be missed, and this is frequently seen in the mesiobuccal (MB) root of maxillary molars [3–5]

One of the main causes of endodontic retreatment is missed canals, with 93% occurring in maxillary first molars and 44% in maxillary second molars [4,6]. The presence of a second mesiobuccal canal (MB2) is a common anatomical variation, with reported incidence rates ranging from 33% to 96% according to various studies in maxillary first molars, whereas maxillary second molars range from 8.5 to 82.6% [7–13]. Various approaches have been employed to identify MB2 canals in maxillary molars in both in vitro and clinical studies. Micro-computed tomography (micro-CT) is considered the gold standard for evaluating root canal anatomy due to its extremely high spatial resolution and ability to provide detailed three-dimensional visualization of internal structures. However, its use is limited to in vitro settings because of high radiation doses, cost, limited field of view, and the need for extracted teeth [14]. In contrast, cone beam computed tomography (CBCT) has been widely used in clinical research and practice since the 1990s for the evaluation of root canal anatomy. CBCT offers several advantages, including a lower effective radiation dose, shorter exposure time, reduced cost compared to conventional CT, adequate spatial resolution for clinical applications, minimal distortion, and nondestructive three-dimensional visualization. Therefore, CBCT was selected in the present study as it provides a practical and reliable method for in vivo assessment of MB2 canals while maintaining clinical applicability [15–17].

Numerous studies have demonstrated the effectiveness of CBCT in detecting a MB2 canal in maxillary first molars (MFMs) and maxillary second molars (MSMs), with detection rates ranging from 69.2% to 96.6% [18,19].

Artificial Intelligence (AI) in endodontics has the potential to improve the diagnosis, treatment, and prevention of pulpal and periapical disease. Advancements in endodontic AI research for clinical applications include the detection and diagnosis of periapical lesions, fractures, resorptions, or root canal anatomy and clinical treatment outcome predictions [20–23].

Machine learning (ML), a key subset of AI, is increasingly being investigated as an adjunctive tool in maxillofacial and dental imaging. In particular, supervised ML approaches were employed, as the outcome of interest was predefined and labeled, which are designed to learn patterns from labeled datasets and generate predictions for new, unseen data, thereby supporting clinical decision-making. Their application has been increasingly reported in endodontics, where they have demonstrated the ability to enhance diagnostic accuracy and consistency compared with conventional assessment methods [24,25]. To ensure robustness and minimize the risk of overfitting, a nested cross-validation strategy was implemented, allowing for optimal model tuning and unbiased performance estimation. This approach strengthens the reliability and generalizability of the predictive models developed in the present study.

CBCT interpretation by clinicians is limited by low inter- and intra-observer agreement, as well as reduced sensitivity and specificity. As a result, AI-based CBCT applications have become increasingly important in minimizing observer bias [26].

This study aimed to determine the incidence of MB2 canals on MFMs and MSMs and to predict its occurrence based on various factors in the Iraqi subpopulation, utilizing CBCTs and a Supervised ML Subset.

## 2. Methods

### 2.1. Study design

The CBCT images utilized in this study were sourced from the archives of the B&R Dental Center in Sulaimani City in the Kurdistan Region of Iraq. The study was approved by the Ethical Committee at the College of Dentistry, University of Sulaimani (D-KA2207). As this was a retrospective study, the requirement for informed consent was waived by the committee. The research adhered to the principles of the Declaration of Helsinki. CBCT images were obtained from 1/7/2025–31/12/2025 for various purposes unrelated to this investigation, and all radiographs were anonymized prior to image analysis, and the data of the study were encrypted to ensure confidentiality and data security.

### 2.2. Sample size calculation

A priori power analysis was performed with G*Power (version 3.1.9.4) to ascertain the minimum necessary sample size. A medium effect size (Cohen’s *d* = 0.5) was presumed, with a significance threshold of α = 0.05 and a statistical power of 95%. The investigation, utilizing a two-tailed independent t-test, revealed that 210 teeth (105 per group) are necessary to identify a statistically significant difference between the two groups.

The study comprised 416 teeth (210 MFMs and 206 MSMs), surpassing the determined sample size criterion. The enlarged sample provided sufficient statistical power for subgroup analysis and enhanced the reliability of the findings.

### 2.3. Study sample

A total of 144 CBCT scans from 63 female and 81 male participants were examined. The participants’ ages varied from 18 to 79 years, with a mean age of 41.75 ± 15.13 years.

In total, 210 MFMs (91 from female subjects and 119 from male subjects) and 206 MSMs (110 from male subjects and 96 from female subjects) were evaluated. All samples met the following inclusion criteria: individuals were required to be above 18 years of age and had at least one completely erupted permanent maxillary first and second molar with a fully developed apex. This allowing for consistent and reliable assessment. Root development in younger patients may be incomplete, which can affect the accuracy of canal visualization. The exclusion criteria encompassed: teeth with fillings or prior root canal treatments, posts, or fixed prosthetic restorations; unclear or incomplete images resulting from scattering or beam-hardening artifacts; and pathological lesions, calcifications, or noticeable root resorption associated with the analyzed tooth.

### 2.4. Image acquisition

All CBCT images were obtained utilizing the Carestream 9600 system (Carestream Dental, Marne-la-Vallée, France). Imaging was acquired with a 10 × 5 cm field of view (FOV) and an isotropic voxel size of 75 µm (75 × 75 × 75 µm). The radiographic parameters were a tube voltage of 120 kV, a current of 8 mA, and an exposure duration of 20 seconds.

Image analysis was performed using CS Imaging 8 software (version 8.0.32; Carestream Dental), which has a reported measurement error margin of approximately 0.14 mm. Images were evaluated in the axial plane using the software’s built-in tools, with contrast and brightness adjusted as needed to optimize visualization.

### 2.5. Morphological analysis and geometric measurement

**Fig 1:**
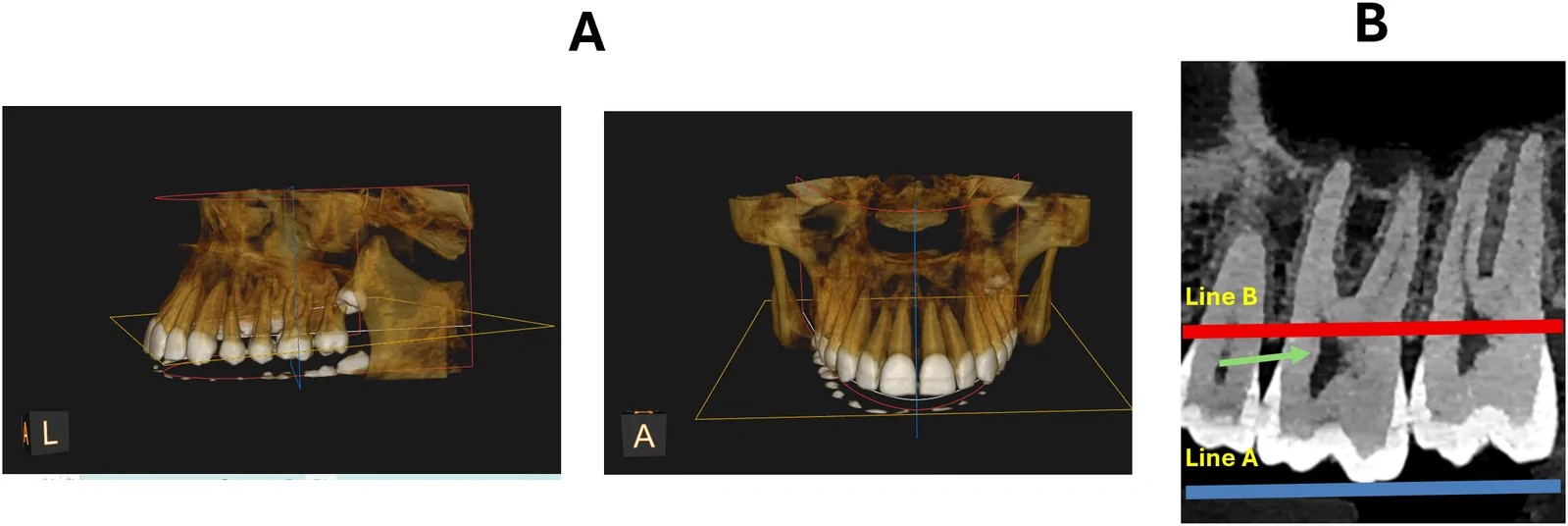
(A) 3D CBCT reconstruction demonstrating standardized orientation for measurements. The horizontal line represents the occlusal reference plane, while the vertical lines correspond to the mid-sagittal and sectional reference planes used for orientation during measurements. The bounding box indicates the defined field of view (region of interest). (B) illustrates the anatomical reference lines used for vertical standardization of measurements on the CBCT image. Line A (blue) represents a horizontal reference line drawn parallel to the cemento-enamel junction (CEJ), serving as the coronal anatomical baseline. Line B (red) is positioned apically, parallel to Line A, at the level corresponding to the pulpal floor region. It also demonstrates the reference points, pulpal floor (green arrow) that were used for analysis in this study.

All eligible images were adjusted to ensure bilateral symmetry of the maxilla, with the occlusal plane, in both frontal and sagittal views, remaining parallel to the ground. The long axis of the examined molar was oriented perpendicular to the ground floor (Fig 1A). The long axis of the analyzed molar was positioned perpendicular to the ground plane. Two horizontal reference lines were then established: Line A, parallel to the inferior margin of the cementoenamel junction, and Line B, aligned with the level of the pulp chamber floor (Fig 1B). The inferior border of the pulpal floor was utilized as the sagittal reference to make the appropriate axial measurement in which all main canal orifices (mesiobuccal (MB), distobuccal (DB), and palatal (P) canals) were simultaneously visible, corresponding to the anatomical pulpal floor. The horizontal distance between the centers of detected orifices was measured at the orifice level [27].

**Fig 2:**
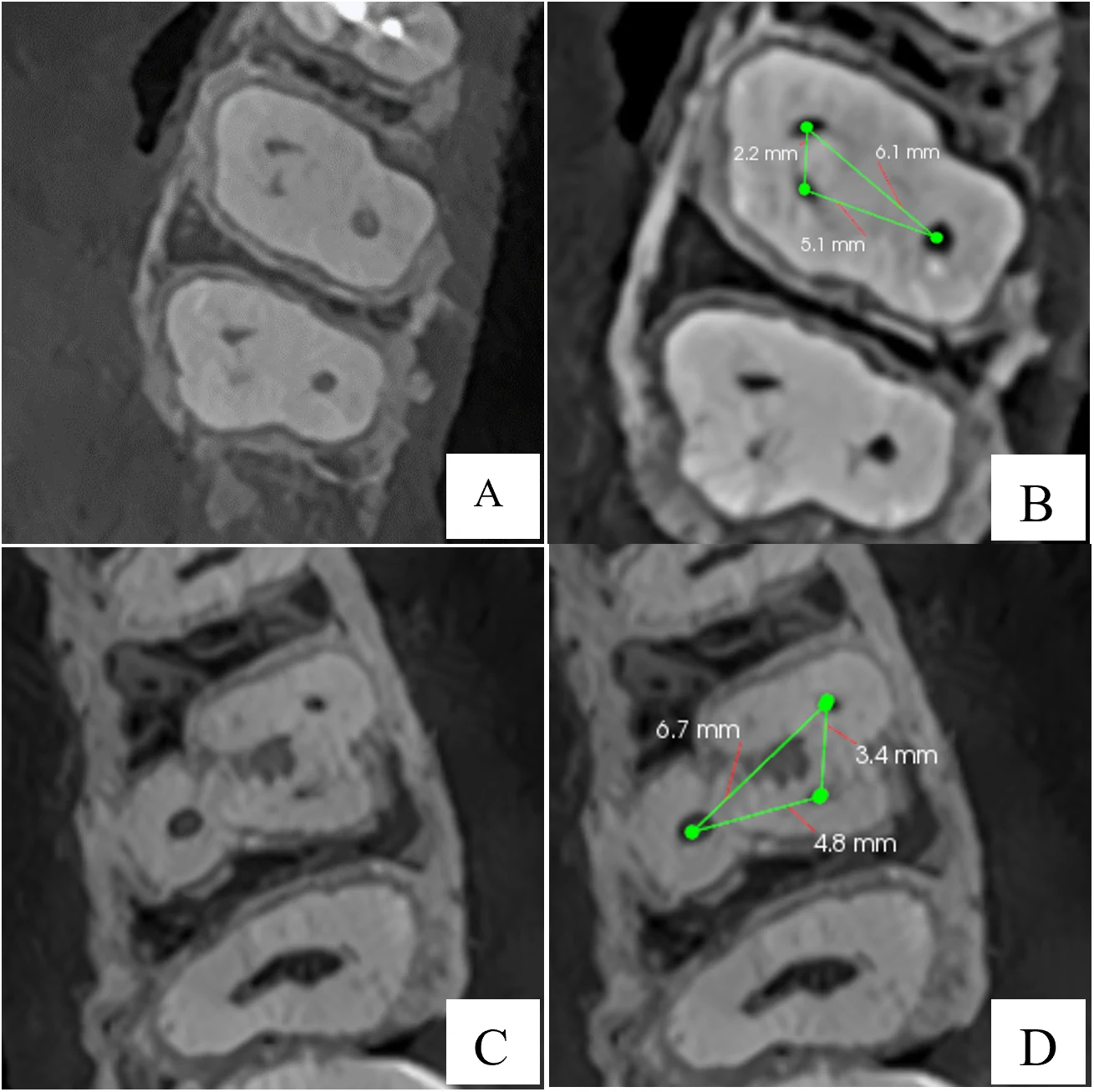
Axial CBCT slices at the level of the pulpal floor showing the initial identification of canal orifices of the MFMs: A: No MB2 canal; B: The interorifice distance measurements between MB, DB, and P canal in absence of MB2 canal; C: with MB2 canal (red arrow); D: The interorifice distance measurements between MB, DB, and P canal in presence of MB2 canal. Note: green points indicate the center of each canal orifice, and the connecting lines represent the linear distances (in millimeters) between canals.

**Fig 3:**
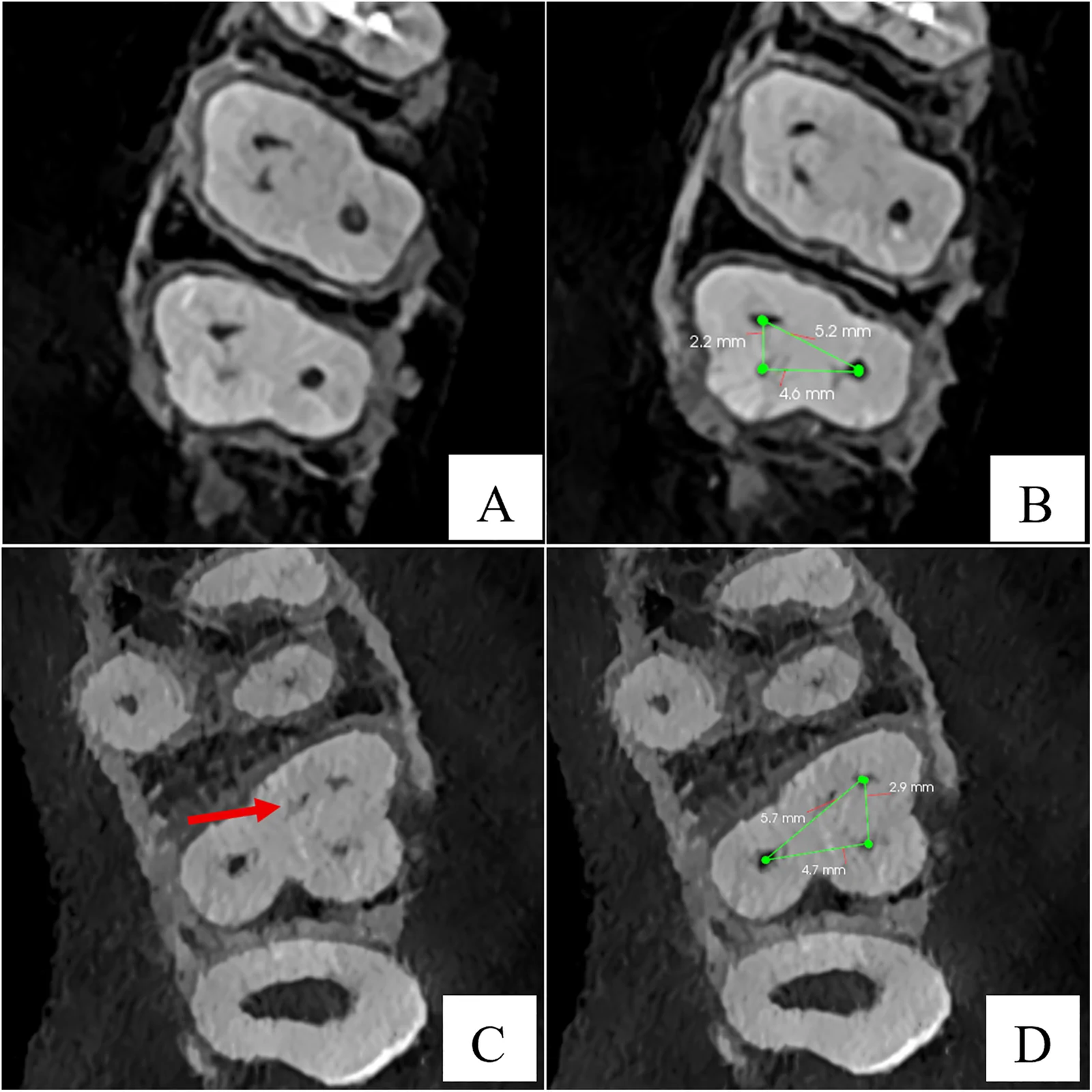
Axial CBCT slices at the level of the pulpal floor showing the initial identification of canal orifices of the MSMs: A: No MB2 canal; B: The interorifice distance measurements between MB, DB, and P canal in absence of MB2 canal; C: with MB2 canal (red arrow); D: The interorifice distance measurements between MB, DB, and P canal in presence of MB2 canal. Note: green points indicate the center of each canal orifice, and the connecting lines represent the linear distances (in millimeters) between canals.


For the interorifice distances (IODs), straight green lines were projected in both MFMs and MSMs in the presence and absence of MB2 canal, joining different points: mesiobuccal to distobuccal (MB-DB) canal, mesiobuccal to palatal (MB-P) canal, and distobuccal to palatal (DB-P) canal (Fig 2 and 3, B and D). The area of the triangle (Area) in millimeter square (mm^2^) of the surface bounded by the central points of each orifice of (MB, DB, and palatal) canals was calculated using Heron’s formula. SP is the semiperimeter of the triangle defined as half the sum of the distances between the MB, DB, and palatal canals, and explain its relevance as a geometric parameter used in triangle-based spatial analysis (e.g., Heron’s formula).

$$\begin{array}{c} Area\;of\;triangle\;(A)=\sqrt{SP(s-a)(s-b)(s-c)} \end{array}$$

where a (distance from MB-DB canals), b (distance from MB-P canals), and c (distance from DB-P canals) are the lengths of the three sides of the triangle; and SP is the semiperimeter (SP) in millimeter (mm) of the triangle defined as SP= (a + b + c)/2).

### 2.6. Reliability test

To assess reliability, an endodontist, highly experienced, evaluated all measurements twice by the same examiner with a four-week interval between sessions, and the mean of the two readings was used for subsequent analysis. Intra-examiner reliability was evaluated using the intraclass correlation coefficient, where values <0.5 indicate poor reliability, 0.5–0.75 indicate moderate reliability, 0.75–0.9 indicate good reliability, and >0.9 indicate excellent reliability [28].

### 2.7. Statistical analysis

All statistical analyses were conducted utilizing the Statistical Package for the Social Sciences Version 25 (IBM Corp., Armonk, NY, USA). Descriptive statistics were computed, encompassing frequency, percentage, mean, standard deviation, median, and interquartile range. The Shapiro–Wilk test was utilized to evaluate the normality of the data. An Independent t-test was employed for parametric data, whilst the Mann–Whitney U test was utilized for non-parametric data.

The receiver operating characteristic (ROC) analysis was performed to ascertain sensitivity, specificity, and cutoff values for evaluating the discriminative efficacy of each parameter in predicting the MB2 canal.

### 2.8. Machine learning models

All ML analyses were implemented in Python (version 3.10; Python Software Foundation, Wilmington, DE, USA) and executed on an MSI GF65 laptop equipped with a 9th-generation Intel® Core™ i7 processor. Six supervised classification algorithms were evaluated: Gaussian Naive Bayes (GNB), Logistic Regression (LR), Decision Tree (DT), Random Forest (RF), Support Vector Classifier (SVC), and K-Nearest Neighbors (KNN).

The dataset split into 80% training data and 20% independent test data using stratified sampling to preserve class distribution. To address class imbalance, the Synthetic Minority Over-sampling Technique (SMOTE) was applied exclusively to the training set, ensuring that the test set remained completely unseen. The nested cross-validation framework was applied on the balanced training subset for training the models and hyperparameter tuning. The outer loop consisted of five stratified folds, in which each fold served once as a validation set while the remaining folds were used for training of the ML models. Performance metrics were averaged across all outer folds.

Following nested cross-validation, each model was retrained on the full balanced training set and evaluated on the held-out 20% test set to provide a final, unbiased assessment of predictive performance. For each classifier, the following evaluation metrics were computed: accuracy, precision, recall, F1-score, and specificity. In addition, confusion matrices, ROC curves were generated, and area under the curve (AUC) values were calculated. Model probability calibration was further assessed using Brier score, log loss, expected calibration error (ECE), maximum calibration error (MCE), and sharpness, with calibration curves plotted for each algorithm.

Finally, feature importance analysis was conducted for the RF and DT models, and the most influential features were visualized to enhance model interpretability.

**Fig 4:**
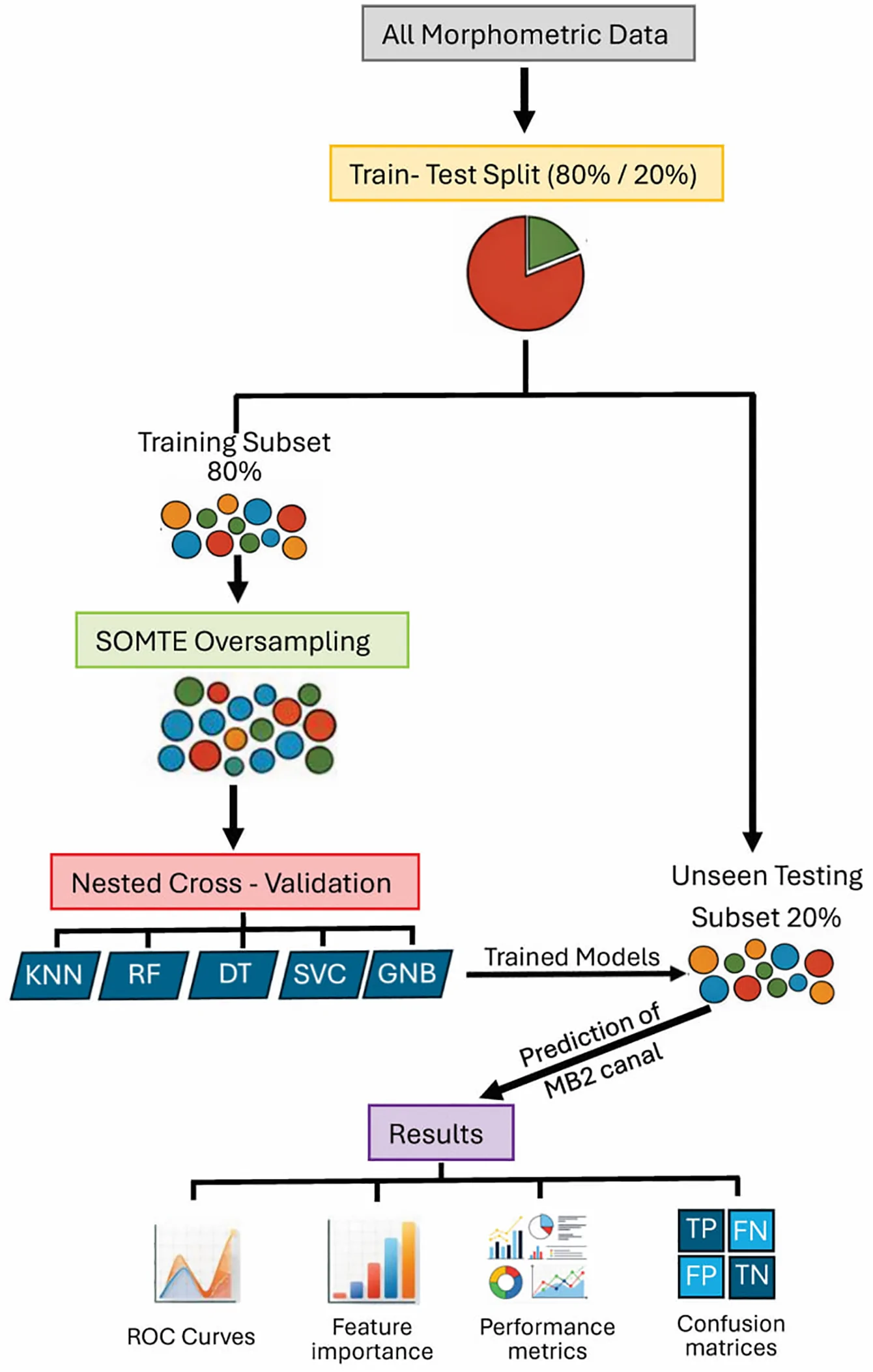
ML workflow of the study.

The ML pipeline, including data splitting, SMOTE oversampling, nested cross-validation, and final model evaluation on an unseen test set, is illustrated in Fig 4.

### 3. Results

In this retrospective study, 144 radiographs from 63 female and 81 male subjects were analyzed. The participants’ ages ranged from 18 to 79 years, with a mean age of 41.75 ± 15.13 years. A total of 210 MFMs were assessed, comprising 91 teeth from female subjects and 119 from male subjects. Additionally, 206 MSMs were examined, including 110 teeth from male subjects and 96 from female subjects.

The reliability analysis indicated excellent intra-examiner consistency, with intraclass correlation coefficient (ICC) values for all evaluated variables ranging from 0.891 to 0.993.

The overall prevalence of the MB2 canal in the MFMs was 65.24%. The highest prevalence was observed in individuals younger than 30 years (73.1%), while the lowest was found in those aged 60 years and older (43.3%); however, this difference was not statistically significant (p = 0.07). No significant associations were found between MB2 prevalence and sex (p = 0.915) or tooth side (p = 0.645) in the MFMs.

Conversely, the prevalence of MB2 in the MSMs was significantly higher among females (51%) than males (24.5%), with a p-value < 0.001. No significant difference was found between the right and left sides in the second molars (p = 0.337). Table 1 summarizes the prevalence of MB2 in both MFMs and MSMs according to age groups, sex, and tooth side.

**Table 1:** Prevalence of MB2 in MFMs and MSMs.

		MB2 Absent No. (%)	MB2 Present No. (%)	Total No. (%)	P value*
MFMs	**Age groups (years)**				
	<30	14 (26.9)	38 (73.1)	52 (100)	0.07
	30-39	16 (29.6)	38 (70.4)	54 (100)	
	40-49	13 (38.2)	21 (61.8)	34 (100)	
	50-59	13 (32.5)	27 (67.5)	40 (100)	
	≥ 60	17 (56.7)	13 (43.3)	30 (100)	
	**Sex**				
	Male	41 (34.5)	78 (65.5)	119 (100)	0.915
	Female	32 (35.2)	59 (64.8)	91 (100)	
	**Side**				
	Right	36 (36.4)	63 (63.6)	99 (100)	0.645
	Left	37 (33.3)	74 (66.7)	111 (100)	
MSMs	<30	19 (44.2)	24 (55.8)	43 (100)	0.049
	30-39	39 (72.2)	15 (27.8)	54 (100)	
	40-49	23 (62.2)	14 (37.8)	37 (100)	
	50-59	27 (65.9)	14 (34.1)	41 (100)	
	≥ 60	22 (71.0)	9 (29)	31 (100)	
	**Sex**				
	Male	83 (75.5)	27 (24.5)	110 (100)	< 0.001
	Female	47 (49.0)	49 (51)	96 (100)	
	**Side**				
	Right	62 (66.7)	31 (33.3)	93 (100)	0.337
	Left	68 (60.2)	45 (39.8)	113 (100)	

*Calculated by the Chi-square test.

Based on the Shapiro–Wilk test, the variables MB-DB, MB-P, DB-P, and SP of the MFMs, as well as MB-DB, MB-P, and DB-P of the MSMs, followed a normal distribution. In contrast, Area of the MFMs, along with Area, and SP of the MSMs, demonstrated non-parametric distributions.

The independent t-test results for all parametric variables and the Mann–Whitney U test results for all non-parametric variables showed no statistically significant differences in the linear measurements of the MFMs and MSMs between the two sexes. Similarly, no significant differences were observed between the left and right sides for all variables, with the exception of MB-P in the MSMs. A comprehensive summary of the results for all studied variables with respect to sex and side, along with the corresponding statistical tests, is provided in Table 2.

**Table 2:** Summary of the statistical analysis of linear measurements of MFMs and MSMs according to sex and side.

Parametric variables		Male Mean ± SD	Female Mean ± SD	P value*	Right side Mean ± SD	Left side Mean ± SD	P value*
**MFMs**	**MB-DB (mm)**	4.232 ± 0.823	4.336 ± 0.847	0.474	4.321 ± 0.889	4.239 ± 0.784	0.574
	**MB-P (mm)**	7.91 ± 1.206	8.173 ± 1.074	0.181	7.919 ± 1.082	8.112 ± 1.213	0.327
	**DB-P (mm)**	6.146 ± 1.245	6.209 ± 1.064	0.753	6.141 ± 1.207	6.2 ± 1.139	0.771
	**SP (mm)**	9.144 ± 1.545	9.359 ± 1.29	0.379	9.191 ± 1.46	9.276 ± 1.432	0.732
**MSMs**	**MB-DB (mm)**	3.611 ± 0.79	3.933 ± 0.743	0.089	3.642 ± 0.824	3.94 ± 0.716	0.108
	**MB-P (mm)**	7.119 ± 1.324	7.512 ± 1.095	0.194	7.016 ± 1.28	7.618 ± 1.066	0.036
	**DB-P (mm)**	5.819 ± 1.402	5.835 ± 1.065	0.959	5.707 ± 1.189	5.913 ± 1.191	0.459
**Non-Parametric variables**		**Male** **Median (IQR)**	**Female** **Median (IQR)**	**P value†**	**Right side** **Median (IQR)**	**Left side** **Median (IQR)**	**P value †**
**MFMs**	**Area (mm**^**2**^)	21.541 (13.12)	21.841 (9.57)	0.971	21.841 (11.31)	21.541 (8.004)	0.964
**MSMs**	**Area (mm**^**2**^)	18.005 (10.47)	18.69 (11.54)	0.614	18.69 (13.53)	18.639 (9.8)	0.186
	**SP (mm)**	8.1 (2.2)	8.45 (2.03)	0.17	8.15 (2.95)	8.45 (1.55)	0.536

* Calculated by Independent t-test, † Calculated by Mann-Whitney U test, SP semiperimeter

All morphometric parameters exhibited statistically significant differences between the MFMs and MSMs with an MB2 canal and those without, except for the Area and DB-P in both molars, which did not show statistically significant differences. Table 3 presents all linear measurements of both molars, comparing teeth with and without an MB2 canal, along with the results of the independent t-test and Mann–Whitney U test.

**Table 3:** Comparison of linear measurements of MFMs and MSMs with and without MB2 canal: Mean ± SD for parametric data and Median (IQR) for non-parametric data, with results of Independent t-test and Mann–Whitney U test.

Parametric variables		MB2	Mean ± SD	P value*
**MFMs**	**MB-DB (mm)**	Absent	3.763 ± 0.972	0.001
		Present	4.277 ± 0.832	
	**MB-P (mm)**	Absent	6.948 ± 1.314	0.001
		Present	8.023 ± 1.154	
	**DB-P (mm)**	Absent	5.877 ± 1.495	0.144
		Present	6.173 ± 1.166	
	**SP (mm)**	Absent	8.294 ± 1.781	0.001
		Present	9.237 ± 1.44	
**MSMs**	**MB-DB (mm)**	Absent	3.259 ± 0.806	0.001
		Present	3.818 ± 0.77	
	**MB-P (mm)**	Absent	6.385 ± 1.22	0.001
		Present	7.372 ± 1.188	
	**DB-P (mm)**	Absent	5.562 ± 1.202	0.123
		Present	5.829 ± 1.187	
**Non-Parametric variables**		**MB2**	**Median (IQR)**	**P value †**
**MFMs**	**Area (mm**^**2**^)	Absent	18.47 (16.56)	0.062
		Present	21.58 (11.32)	
**MSMs**	**SP (mm)**	Absent	7.4 (2.33)	0.001
		Present	8.3 (1.89)	
	**Area (mm**^**2**^)	Absent	16.4(11.77)	0.053
		Present	18.67 (10.85)	

* Calculated by Independent t-test, † Calculated by Mann-Whitney U test, SP semiperimeter

The results of the ROC analysis demonstrated that the SP parameter exhibited the highest discriminative ability for detecting the presence or absence of the MB2 canal in MFMs with AUC of 0.665, while MB-P distance exhibit the highest discriminative ability with AUC values of 0.723 for MSMs. In contrast, the DB-P distance parameter showed the lowest discriminative power among all measured variables, with AUC values of 0.572 for the MFMs and 0.565 for the MSMs.

**Fig 5:**
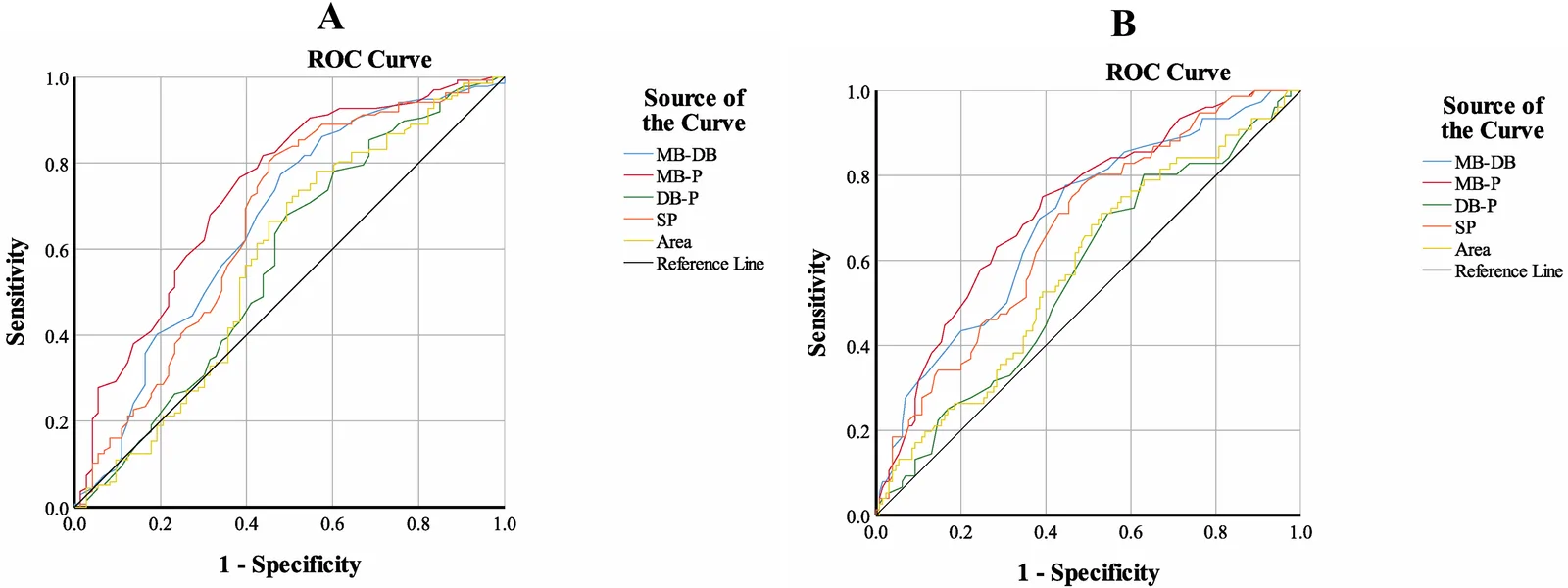
The ROC curve of all linear and angular parameters: A. MFMs; and B. MSMs.

Table 4 summarizes the AUC values, optimal cut-off points, sensitivity, and specificity for all assessed parameters in both molars. The ROC curves for these parameters are illustrated in Fig 5.

**Table 4:** ROC analysis of morphometric parameters for predicting the presence of MB2 canal in MFMs and MSMs: AUC, cut-off value, sensitivity, and specificity.

Tooth	Parameter	AUC (95% CI)	Cut-off value	P value	Sensitivity	Specificity
**MFMs**	**MB-DB**	0.664 (0.583 - 0.745)	4.05	0.001	0.62	0.603
	**MB-P**	0.663(0.736 - 0.809)	7.55	0.001	0.679	0.685
	**DB-P**	0.572 (0.486 - 0.658)	5.95	0.086	0.54	0.562
	**SP**	0.665 (0.583 - 0.747)	8.875	0.001	0.599	0.616
	**Area**	0.578 (0.492 - 0.665)	20.755	0.062	0.577	0.575
**MSMs**	**MB-DB**	0.695 (0.621- 0.768)	3.65	0.001	0.618	0.659
	**MB-P**	0.723 (0.652 - 0.793)	6.95	0.001	0.658	0.667
	**DB-P**	0.565 (0.485–0.646)	5.65	0.118	0.539	0.558
	**SP**	0.674 (0.6 - 0.748)	8.075	0.001	0.618	0.628
	**Area**	0.583 (0.503 - 0.664)	17.725	0.046	0.553	0.558

For MFMs, after splitting the data the training subset consisted of 58 cases without MB2 canal and 110 cases with MB2 canal. After applying SMOTE, the dataset was balanced, resulting in 110 cases for both with and without MB2 canal. For MSMs, the training subset included 103 cases with an MB2 canal and 61 cases without. Following SMOTE, the sample was balanced to 103 cases for both groups.

The performance of the trained with nested cross-validation ML models evaluated on the testing subset of MFMs for the prediction of MB2 revealed that the LR model outperformed all other models with an accuracy of 0.762, precision of 0.785, recall of 0.762, F1-score of 0.766, and specificity of 0.8. In contrast, the GNB model demonstrated the poorest performance in predicting MB2, yielding an accuracy of 0.667, precision of 0.667, recall of 0.667, an F1-score of 0.667, and specificity of 0.533.

On the other hand, analysis of the testing subset using ML models trained with nested cross-validation on the morphometric parameters of MSMs demonstrated substantially higher predictive performance for MB2. Among these models, LR achieved the best results, with an accuracy of 0.833, precision of 0.837, recall of 0.833, F1-score of 0.834, and specificity of 0.852. The detailed performance metrics, including accuracy, precision, recall, F1-score, and specificity for all evaluated ML models, are presented in Table 5.

**Table 5:** Performance metrics of all ML models in the analysis of morphometric parameters of MFMs and MSMs for the prediction of the MB2 canal.

Tooth	Model	Accuracy	Precision	Recall	F1-score	Specificity
**MFMs**	RF	0.738	0.735	0.738	0.736	0.6
	DT	0.69	0.686	0.690	0.688	0.533
	SVC	0.714	0.724	0.714	0.718	0.667
	KNN	0.69	0.707	0.69	0.695	0.667
	GNB	0.667	0.667	0.667	0.667	0.533
	LR	0.762	0.785	0.762	0.766	0.8
**MSMs**	RF	0.762	0.762	0.762	0.762	0.815
	DT	0.667	0.658	0.667	0.661	0.778
	SVC	0.738	0.769	0.738	0.743	0.704
	KNN	0.714	0.714	0.714	0.714	0.778
	GNB	0.69	0.722	0.69	0.697	0.667
	LR	0.833	0.837	0.833	0.834	0.852

**Fig 6:**
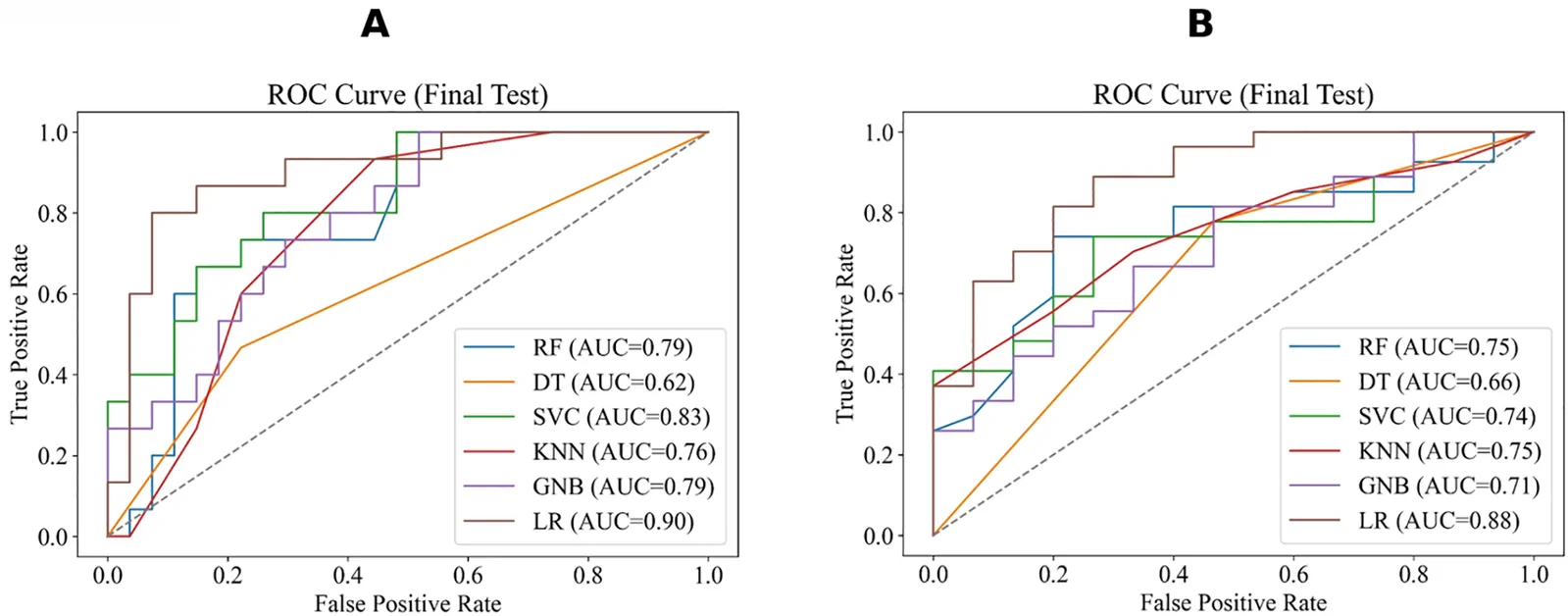
The results of the ROC analysis of all ML Algorithms, A. MFMs; and B. MSMs.

**Fig 7:**
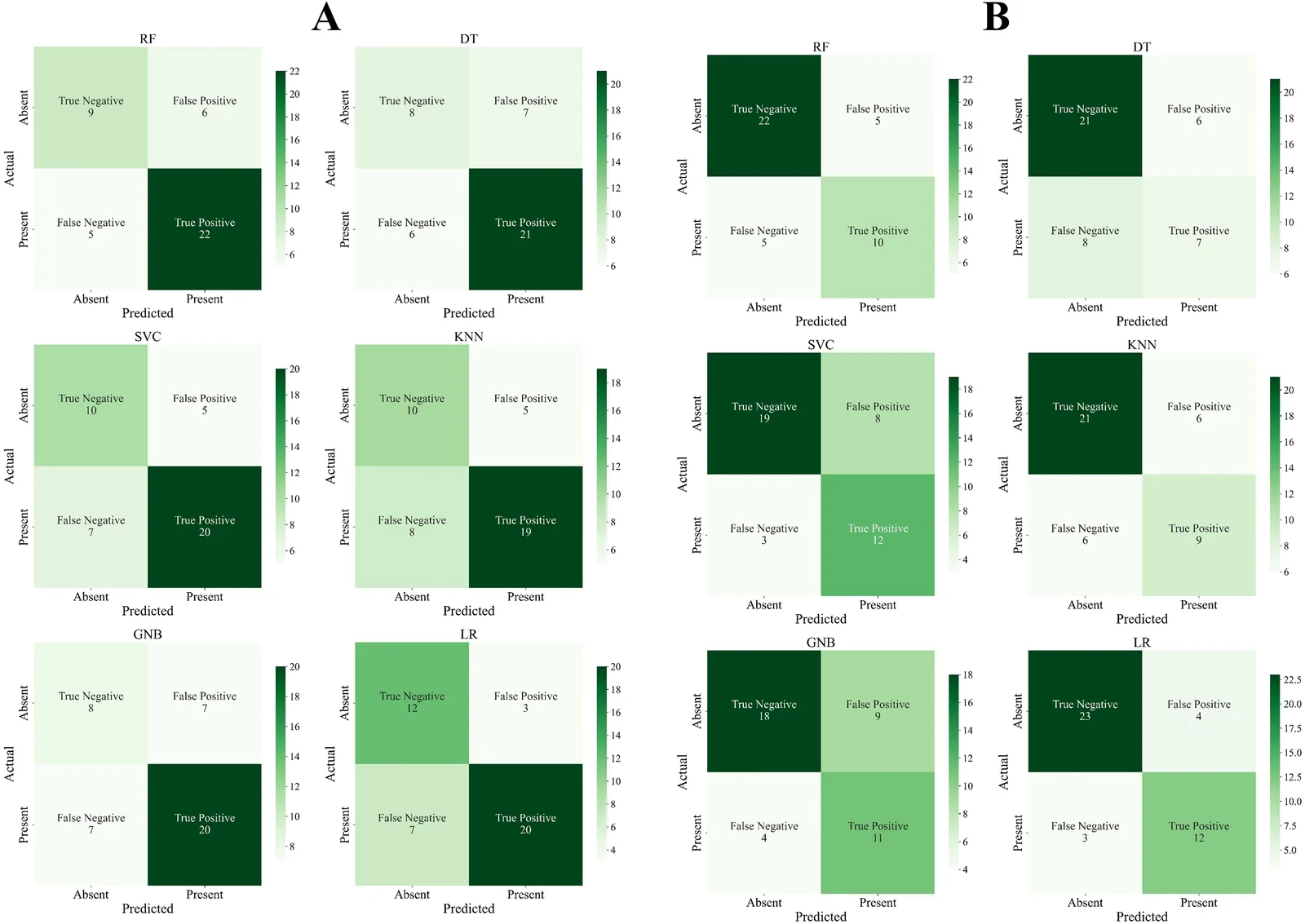
Confusion matrices of all ML models. A. MFMs; and B. MSMs.

The AUC values of the ML models ranged from 0.66 to 0.88, with LR achieving the highest AUC for the prediction of MFMs. Similarly, LR demonstrated the best performance among all ML classifiers for MSMs parameters, yielding an AUC of 0.90. Analysis of the confusion matrix for the LR model revealed 3 false positives and 7 false negatives for MFMs, and 4 false positives and 3 false negatives for MSMs. As shown in Fig 6, the ROC curves and corresponding AUC values of all ML models are presented for both molar types, while Fig 7 illustrates the confusion matrices of all evaluated ML models.

In predicting MB2 in the MFMs, the LR model achieved the best overall probabilistic performance, with the lowest Brier score and log loss. The SVC and RF models showed moderate calibration and acceptable sharpness. In contrast, decision tree and GNB models demonstrated higher calibration errors and uncertainty, indicating inferior reliability.

**Fig 8:**
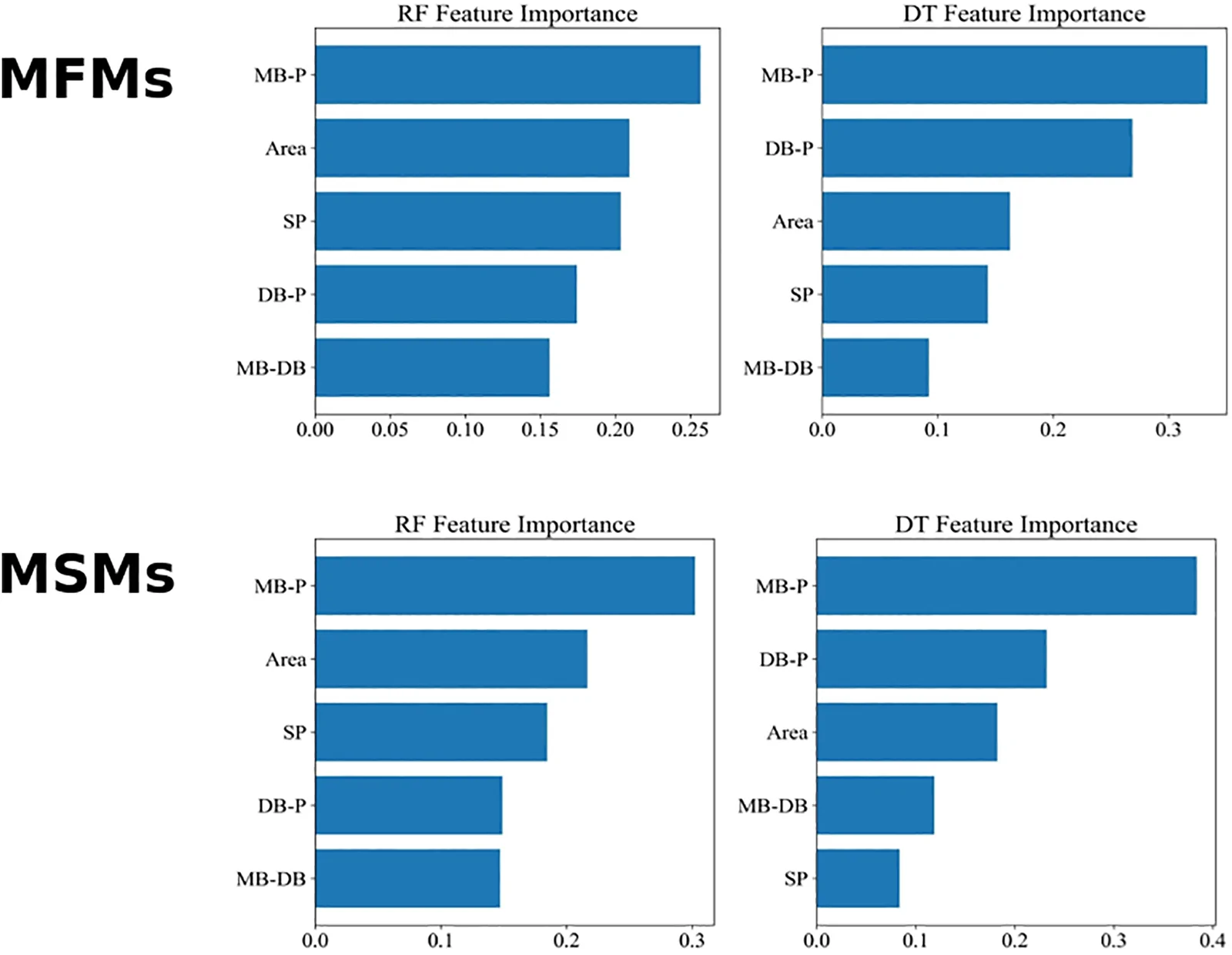
Feature importance derived from RF and DT models for predicting the presence of the MB2 canal in both MFMs and MSMs.

Similarly, in predicting MB2 in the MSMs, the LR model demonstrated superior probabilistic performance, achieving the lowest Brier score, log loss, and calibration errors, reflecting highly reliable risk estimation. The SVC model also showed acceptable calibration and low sharpness. Conversely, DT and KNN exhibited substantial miscalibration and high uncertainty, limiting their clinical applicability. All results of calibration analysis are presented in Table 6, while Fig 8 shows the feature importance of DT and RF regarding prediction of MB2 canal in both MFMs and MSMs.

**Table 6:** Calibration metrics of ML models for prediction of MB2 canal in both MFMs and MSMs.

Model		Brier Score	Log Loss	ECE	MCE	Sharpness
**MFMs**	**RF**	0.196	0.582	0.246	0.65	0.081
	**DT**	0.31	10.691	0.25	0.25	0.222
	**SVC**	0.216	0.625	0.194	0.264	0.036
	**KNN**	0.214	2.112	0.095	0.175	0.11
	**GNB**	0.262	0.982	0.366	0.629	0.143
	**LR**	0.155	0.465	0.19	0.536	0.122
**MSMs**	**RF**	0.183	0.584	0.241	0.99	0.09
	**DT**	0.333	11.513	0.462	0.462	0.214
	**SVC**	0.176	0.533	0.148	0.261	0.033
	**KNN**	0.198	1.326	0.297	1	0.077
	**GNB**	0.208	0.602	0.221	0.475	0.116
	**LR**	0.125	0.401	0.122	0.232	0.08

## 4. Discussion

The aim of the present study was to investigate the application of ML in predicting MB2 canals in both MFMs and MSMs using the CBCT.

Previous studies have reported that maxillary molars exhibit the highest incidence of missed canals during endodontic treatment, with a prevalence of approximately 55%. Additionally, in cases of endodontically treated teeth with untreated canals, apical periodontitis has been observed in up to 96.55% of cases [5,29].

CBCT imaging was used in this study to calculate the morphometric measurement of MB, DB, and P canals in the absence and presence of MB canals in both MFMs and MSMs. CBCT is a non-invasive, precision three-dimensional technique that increases the percentage of therapeutic success and showed superiority over conventional X-rays in detecting the presence of accessory channels [30]. It is as a more reliable method for the detection of the MB2 canal compared to the gold standard of physically sectioning the specimen [31].

A systematic review and meta-analysis by Sadr et al. analyzing pooled data from 12 studies reported sensitivity values for radiographic periapical lesion detection ranging from 0.65 to 0.96—comparable to the diagnostic accuracy of human clinicians using CBCT [32].

The MB2 canals in maxillary molars are often not visible on conventional radiographs, making them susceptible to being missed or improperly managed by clinicians. In such cases, CBCT can provide a more accurate diagnosis when an MB2 canal is suspected. The location and negotiation of the MB2 canal can be significantly aided by CBCT, offering detailed information regarding the direction and position of the root canal [33]. The incidence of MB2 canals in MFMs and MSMs varies from 8.0% to 96.1% [34–36]. In this study, the prevalence of MB2 was 65.24% in MFMs and 36.89% in MSMs, which is similar to findings from other studies [9,37,38].

It was found that presence of MB2 canals in both MFMs and MSMs is not significant in relation to age, sex and sides except in MSMs, where they are significantly present in females compared to males in the current study. Other studies have also found no differences between genders in the presence of MB2 canal [17,39,40].

These variations may be attributed to differences in sample size, methodology, and ethnic background. Additionally, discrepancies in results obtained from CBCT imaging could be due to variations in CBCT parameter settings and software used for analysis [34]. Detecting MB2 canals can be particularly challenging, as the orifice is often covered by a dentin cap [41]. Furthermore, some MB2 canals exhibit curvatures, with their coronal portions containing one or more abrupt bends. These anatomical complexities likely contribute to the higher detection rates of MB2 canals in in vitro studies compared to in vivo studies [42].

In the present study, it was found that with age progression, the prevalence of MB2 decreased. The incidence of MB2 in both MFMs and MSMs was more prevalent in individuals under 30 years old and the lowest percentage was found in those aged 60 years and older, which is consistent with the results of Magat & Hakbilen [43], Betancourt et al. [37] and Zheng et al. [44] and this may be related to an increase in the canal calcification, tertiary dentin formation and porosity of the cortical bone [45–47].

This study focuses on the occurrence and precise position of MB2 canals; the MB2 orifice consistently presents in a mesial and palatal direction relative to the MB1 orifice in both MFMs and MSMs.

We observed that in the MFMs, the distance of MB-DB, MB-P, DB-P canals in the absence of MB2 canal was (3.763 ± 0.972, 6.948 ± 1.314, and 5.877 ± 1.495) and (4.277 ± 0.832, 8.023 ± 1.154, and 6.173 ± 1.166) in the presence of MB2 canal. While in MSMs, the distance was (3.259 ± 0.806, 6.385 ± 1.22, and 5.562 ± 1.202) of MB-DB, MB-P, and DB-P canals in the absence of MB2 canal and (3.818 ± 0.77, 7.372 ± 1.188, 5.829 ± 1.187) in the presence of MB2 canal. The finding of this study is consistent with other studies [19,27,37].

In the present study, Angular parameters (MB angle, DB angle, Palatal angle) are not measured as it is highly influenced by CBCT slice orientation, crown rotation, individual anatomical variation, angles vary widely even among teeth with confirmed MB2 and generally not significant predictors in multivariate regression analyses [48,49].

AI refers to the simulation of human intelligence processes by computer systems, enabling machines to perform tasks that typically require human cognition, such as learning, reasoning, and decision-making [50]. A wide array of areas has been explored regarding possible application of AI in endodontics, which include tooth segmentation, detection of caries, periapical pathologies, obturation, and C-shaped canals [51,52].

Recently, various machine learning algorithms have been employed to identify and categorize dental morphological abnormalities, as well as to discover and localize MB2 canals in maxillary molars [52–54].

A crucial factor affecting the efficacy of a deep learning model is the sample size. Fang et al. [54] evaluated the optimal sample size required to train a deep learning algorithm for segmenting multiple regions of interest in the head and neck, such as the optic nerves and the lens of the eye, and found that 200 samples were needed to achieve 95% effectiveness. Given the complexity of the segmentation task and the relatively small region of interest, in the present study, to predict the presence of the MB2 canal, we used sample sizes of 210 MFMs and 206 MSMs.

In the present study, we measured distances between (MB-DB, MB-P and DB-P) canals, semiperimeter, and area of triangle between (MB, DB and P) canal in the presence and absence of MB2 canal in both MFMs and MSMs on CBCT images, and then we used 6 different ML algorithms to predict MB2 canals in MFMs and MSMs.

Based on available information, this is the first study to apply ML to predict the MB2 canal in both MFMs and MSMs using CBCT images. This work employed various ML techniques to address the constraints of discriminant function analysis from CBCT in identifying the MB2 canal in both MFMs and MSMs. The sample was divided, allocating 80% for training and 20% for testing the models.

LR achieved the highest AUC (0.88) for predicting MFMs among all ML models. Similarly, LR demonstrated the best performance for MSMs, with an AUC of 0.90. Comparable accuracy was reported for ML-based detection of MB2 canals in maxillary molars using axial CBCT slices [55]. The findings of the present study are consistent with those of Shetty et al. [55], who reported that ML algorithms showed strong performance in detecting MB2 canals using axial CBCT images, as demonstrated by their AUC, precision, and recall values.

Another study, by Karataş et al. [56], reported that in the binary classification task, the system correctly identified 502 out of 526 root canal orifices in MFMs and MSMs, yielding an accuracy of 91%. The YOLO-based CNN demonstrated high accuracy and sensitivity in detecting root canal orifices and classifying orifices into categories such as MB1, MB2, DB, and P from dental operating microscope images.

In all models from ML, feature importance derived from RF and DT models for predicting the presence of the MB2 canal in both molars demonstrated superior probabilistic performance in predicting MB2 canal in both MFMs and MSMs based on distance from MB-P canal.

In this study, linear inter-orifice MB-P distance in both MFMs and MSMs was higher in presence of MB2 canal (8.023 ± 1.154) in MFMs and (7.372 ± 1.188) in MSMs while in the absence of MB2 canal was (6.948 ± 1.314) in MFMs and (6.385 ± 1.22) in MSMs revealed that MB-P distance had accepted diagnostic accuracy for predicating MB presence. This finding agrees with other results [11,49,57].

Application of ML models in this study for predicting MB2 canals in MFMs and MSMs can provide objective, consistent, and reliable predictions, reducing inter-rater variability and enhancing diagnostic accuracy, especially in challenging cases where MB2 canals may be missed. Additionally, ML can serve as a decision support tool, streamlining the diagnostic process and reducing clinician workload by prioritizing cases that require closer attention. Furthermore, ML models could be integrated into clinical settings where CBCT images are not available or in cases of limited access to specialists, providing predictive capabilities using alternative imaging modalities. Ultimately, the application of ML in this context has the potential to improve diagnostic efficiency, reduce human error and support clinicians in making more informed decisions [55,58,59].

This research possesses multiple limitations. First, limited sample size for ML modeling. A future studies with larger, more diverse datasets would help to validate and enhance the robustness of the current finding. Second, all sample teeth were obtained from an Iraqi subpopulation; hence, caution is advised when generalizing the findings to other ethnic or geographical cohorts. Additional research including many demographics is required to substantiate these findings. Third, all CBCT measurements were performed by a single operator, which may introduce operator-dependent variability and potential subjectivity, despite high intra-observer reliability confirmed by ICC analysis. Lastly, while all data were de-identified and handled in accordance with institutional protocols, we recognize the inherent need to maintain strict compliance with data protection regulations, including General Data Protection Regulation (GDPR), to safeguard patient confidentiality. These limitations should be considered when interpreting the results and planning future studies with larger, multi-center cohorts and multiple operators to enhance reproducibility and generalizability.

From a clinical perspective, the findings of the present study demonstrate that the integration of ML following CBCT interpretation enables accurate prediction of MB2 canal occurrence based on the linear measurement of the MB-P distance in both MFMs and MSMs. This predictive approach offers significant clinical value by minimizing the risk of undetected canal anatomy and thereby reducing the potential for endodontic treatment failure.

## 5. Conclusion

The present study can provide a base model for applying ML models that can predict presence of MB2 canals in CBCT radiographs of both MFMs and MSMs. MB-P distance parameter is considered the best indicator to predict the presence of the MB2 canal in both PMFMs and PMSMs.

The identification of the MB-P distance as the most influential predictor provides a tangible, quantifiable parameter that can be incorporated into educational training, assisting dental students and clinicians in understanding key anatomical relationships and improving their diagnostic skills. Overall, the study highlights both a clinical decision-support tool and an educational resource for enhancing endodontic practice.

## Abbreviations

3D: Three-dimensional
A: Area
AI: Artificial Intelligence
AUC: Area under the curve
CBCT: Cone-beam computed tomography
cm: Centimetre
CT: Computed tomography
DB: Disto-buccal
DT: Decision tree
ECE: Expected calibration error
GDPR: General Data Protection Regulation
GNB: Gaussian Naive Bayes
IODs: Interorifice distances
KNN: K-Nearest Neighbors
LR: Logistic regression
MB: Mesio-buccal
MB-P: Mesiobuccal to palatal
MB2: Second mesiobuccal
MCE: Maximum calibration error
MFMs: Permanent maxillary first molars
MSMs: Permanent maxillary second molars
ML: Machine learning
mm: Millimetre
mm^2^: Millimetre square
P: Palatal
RF: Random forest
ROC: Receiver operating characteristic
SMOTE: Synthetic Minority Over-sampling Technique
SP: Semiperimeter
SVC: Support Vector Classifier
μm: Micrometre
